# Kinase Signaling in Dendritic Development and Disease

**DOI:** 10.3389/fncel.2021.624648

**Published:** 2021-02-10

**Authors:** Kimya Nourbakhsh, Smita Yadav

**Affiliations:** Department of Pharmacology, University of Washington, Seattle, WA, United States

**Keywords:** dendrites, kinases, neurodevelopmental diseases, neurological disorder, kinome

## Abstract

Dendrites undergo extensive growth and remodeling during their lifetime. Specification of neurites into dendrites is followed by their arborization, maturation, and functional integration into synaptic networks. Each of these distinct developmental processes is spatially and temporally controlled in an exquisite fashion. Protein kinases through their highly specific substrate phosphorylation regulate dendritic growth and plasticity. Perturbation of kinase function results in aberrant dendritic growth and synaptic function. Not surprisingly, kinase dysfunction is strongly associated with neurodevelopmental and psychiatric disorders. Herein, we review, (a) key kinase pathways that regulate dendrite structure, function and plasticity, (b) how aberrant kinase signaling contributes to dendritic dysfunction in neurological disorders and (c) emergent technologies that can be applied to dissect the role of protein kinases in dendritic structure and function.

## Introduction

Dendrites are specialized neuronal processes that receive and integrate synaptic or sensory input. While dendrites are extremely heterogeneous morphologically, dendrites of a certain neuron type generally exhibit stereotyped morphology (Jan and Jan, 2001). Dendrites are sculpted by intrinsic genetic programs, external cues, and patterns of neuronal activity during development (Cline, 2001; Jan and Jan, 2010). These factors together confer dendrites their type specific morphology, and ensure fidelity in forming synaptic connections. Owing to the critical role dendrites play in establishing neuronal connectivity, their dysfunction is strongly associated with several neurological disorders (Forrest et al., 2018). Over the last four decades, multiple molecular signaling pathways that mediate structural and functional development of dendrites have been identified. Protein kinases play a pivotal role in almost all aspects of dendritic development and function, while their dysregulation contributes extensively to disease states.

Kinases catalyze the transfer of a phosphate group from ATP molecule to hydroxyl group containing amino acids primarily serine, threonine and tyrosine on substrate proteins, in a process termed as phosphorylation (Cohen, 2002; Fabbro et al., 2015). Several characteristics make kinase signaling uniquely powerful and versatile: (1) Phosphorylation occurs in a highly specific yet reversible fashion. (2) Phosphorylation can affect substrates in distinct ways: such as induce a gain or loss of substrate activity, change substrate localization or interactome. (3) Kinases often act in cascades and are capable of remarkable signal integration and amplification that can be tuned to achieve a variety of responses (Cobb, 1999). (4) Kinases are highly druggable making them promising therapeutic targets (Cohen, 2002). Several protein kinases are genetically linked to neurological disorders. These include neurodevelopmental disorders such as autism spectrum disorder as well as neurodegenerative diseases such as Parkinson's disease, yet little is known about how dysfunction in kinase signaling leads to pathological states (Baltussen et al., 2017; Krahn et al., 2020). Phosphorylation mediated by kinases is countered by phosphatases, a large family of enzymes that catalytically remove phosphate groups from their substrates (Barford et al., 1998; Peng and Maller, 2010). Phosphatases are divided into subfamilies of serine/threonine and tyrosine phosphatases, and dysfunction of these enzymes is associated with several diseases (Tonks, 2006). Activity of phosphatases is tightly regulated by diverse mechanisms that include binding with inhibitory proteins, direct oxidation, and kinase mediated phosphorylation (den Hertog, 2003). Thus, the true phosphorylation status of a protein in time and space is determined by the opposing action of protein kinases and phosphatases.

Herein, we describe how kinase signaling exquisitely orchestrates each step of dendrite development, beginning at neurogenesis to its maturation into synaptic networks. Evidence for critical and causative role of kinase dysfunction in neurodevelopmental and degenerative disorders is presented. Finally, we detail the emergent technologies that will be instrumental in delineating kinase function in dendritic development and how kinase dysfunction leads to pathologies associated with neurological disorders.

### Kinase Pathways That Regulate Dendrite Structure, Function and Plasticity

Before the inception of neurogenesis, the developing brain is comprised of polarized neuroepithelial cells that line the neural tube (Martin-Belmonte and Mostov, 2008; Taverna et al., 2014). Apical-basal polarity is established early in the developing brain, and is achieved through junctional complexes and polarized protein trafficking. These are regulated by kinase signaling through the conserved Par complex. The Par complex is comprised of scaffolding proteins Par3 and Par6, and the kinase atypical protein kinase C (aPKC) that together establish the apical-basal polarity (McCaffrey and Macara, 2012). The Rho GTPase Cdc42 targets and activates the Par complex at the apical membrane, which is separated from the basolateral membrane domain by adherens junctions. In the mammalian neocortex, intrinsic neuroepithelial polarity set up by the Par complex asymmetrically orients the mitotic spindle, that remarkably, during cell cycle can give rise to asymmetric division (Rodríguez-Fraticelli et al., 2011; Lancaster and Knoblich, 2012). Radial glial cells arising from the asymmetrical division of polarized neuroepithelial cells can further differentiate into neural progenitor cells (NPCs) or neurons through asymmetrical division (Götz and Huttner, 2005). The arising NPCs further self-renew or undergo a terminal differentiation into neurons (Florio and Huttner, 2014). Radial glial cells retain the epithelial polarity set up by the Par complexes. Knockout of one of the isoforms of aPKC, aPKC-λ, in neuroepithelial cells and radial glial cells results in the loss of apical processes that cause disordered layering of the cortex, highlighting the role of aPKC in apical-basal polarity (Imai et al., 2006). Apically located Par complex promotes self-renewing of progenitors at the expense of neurogenic differentiation in the developing cerebral cortex (Costa et al., 2008; Sottocornola et al., 2010). While the Par complex proteins set up intrinsic polarity, extrinsic cues greatly affect neuronal polarity, migration and layer formation in brain development. Secreted factors such as reelin, semaphorins, and neurotrophic factors play important roles in instructing neuronal polarity and migration during early development, and are executed by distinct kinase pathways. We direct readers to in depth reviews detailing how these extrinsic cues and growth factors regulate early brain development (Huang and Reichardt, 2001; Yazdani and Terman, 2006; Jossin, 2020). Here we will focus primarily on the kinase signaling pathways important for dendritic growth, structure and functional maturation.

### Dendritic Arborization: Kinases That Regulate Dendritic Growth, Branching, and Tiling

**Figure 1:**
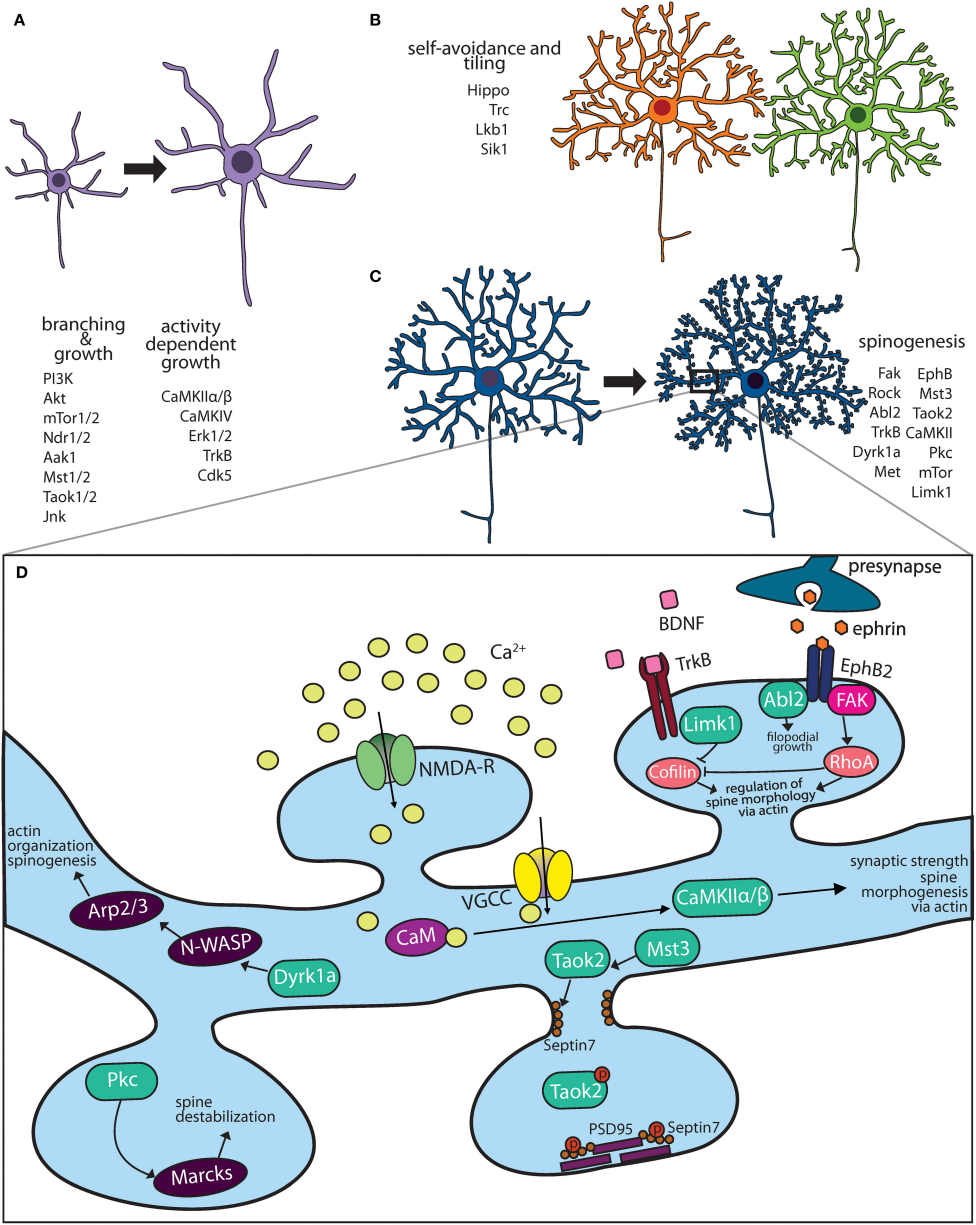
Kinase pathways mediate dendritic morphogenesis and maturation. **(A)** Immature dendritic neurites undergo expansive growth and branching during early development. The PI3K-Akt-mTor kinase and Hippo pathway are critical for growth. Activity dependent growth is primarily mediated by Ca^+2^ influx and downstream signaling by CaMK family members. **(B)** During arborization, dendrites of the same neuron avoid overlap through a principle known as self-avoidance, while neighboring neurons of the same type grow in well-defined territories. Kinases that mediate self-avoidance and dendritic tiling have been identified through screens in Drosophila peripheral sensory da neurons. **(C)** Most excitatory neurons on maturation form actin rich protrusions called dendritic spines that serve as sites of synapse formation. Several kinases that regulate the synaptic cytoskeleton are important regulators of spine formation in hippocampal and cortical neurons. **(D)** Calcium influx through N-methyl D-aspartate (NMDA) receptors and voltage gated calcium channels (VGCC) activate CaMK kinases, which mediate spine morphogenesis and plasticity. Mutations in several kinases important for dendritic spine formation are associated with neuropsychiatric diseases.

Most newly generated mammalian neurons migrate from the site of neurogenesis to their final destination where they are integrated into neural networks. It is during this migration process that they acquire axon-dendrite polarity (Polleux and Snider, 2010). Some neurons, such as retinal ganglion cells, acquire the polarity of the progenitors from which they arise. Others, such as cortical pyramidal neurons, consolidate multiple extended neurites into one leading and one lagging neurite that gives rise to axo-dendritic polarity (Barnes and Polleux, 2009; Polleux and Snider, 2010). On reaching their final destination, neurons extend their dendrites through growth, dendrites scale in size with organism development, and mature in an exquisitely controlled fashion. Control of dendritic development in response to neuronal activity and neurotrophic factors is mediated by kinases, which play an instrumental role in regulating dendritic size and maturation to form synaptic contacts (Figures 1A–D).

#### Kinases as Biochemical Switches for Activity Dependent Dendritic Development

Neuronal activity during brain development profoundly impacts dendritic growth and retraction (Cline, 2001). Experiments where neuronal activity is manipulated such as in sensory deprivation (Wiesel and Hubel, 1963), pharmacological block of activity in *Xenopus* tadpoles (Rajan and Cline, 1998), or enhanced environmental enrichment in rodents (Volkmar and Greenough, 1972), induces dramatic alteration in both dendritic development and its structural complexity. Calcium influx in response to neuronal activity leads to kinase activation, providing the biochemical signal that mediates activity dependent dendritic growth dynamics (Ghosh and Greenberg, 1995). Calcium enters through glutamate receptor NMDA or voltage gated calcium channels (VGCC) and is sequestered by the calcium binding protein, calmodulin. In the Ca^+2^ bound state, calmodulin binds calcium/calmodulin-dependent protein kinases (CaMK), which then undergo autophosphorylation mediated activation. Several CaMKs have been implicated in dendritic development, and these act either *via* local effects or though transcriptional changes (Redmond and Ghosh, 2005). CaMKII has been extensively studied, most famously in relation to dendrite development and synaptic long-term potentiation. Two isoforms of CaMKII are expressed in the brain, CaMKIIα and CaMKIIβ, which each mediate distinct roles in dendrite development in hippocampal neurons, primarily on account of their differential ability to associate with actin (Shen et al., 1998). CaMKIIα expression in tectal neurons in tadpoles was found to stabilize dendritic arbors, as premature expression of CaMKIIα causes dendrites to slow their growth rate to that of more mature neurons. Conversely, blocking endogenous CaMKII maintains neurons in their rapid growth phase, such that dendritic arbors grow larger than normal (Wu et al., 1999; Zou and Cline, 1999). CaMKIIβ, on the other hand promotes actin polymerization, thereby increasing filopodial extension and growth of fine dendrites in rat hippocampal neurons (Fink et al., 2003). Another member of the CaM kinase family CaMKIV, is also crucial for activity dependent dendritic growth. Blocking CaMKIV signaling reduces calcium-induced dendritic growth, while expression of an activated form of CaMKIV in rat cortical neurons mimics the dendrite growth induced by calcium influx (Redmond et al., 2002). CaMKIV is enriched in the nucleus and its effect on dendrite growth is primarily mediated through transcriptional changes. Interfering with activity of transcriptional targets of CaMKIV blocks the ability of active CaMKIV to induce dendrite growth (Redmond and Ghosh, 2005). Importantly, activity induced changes in dendritic growth mediated by CaMKs are developmentally regulated. While the peak expression of CaMKIIβ and CaMKIV coincides with the period of maximal dendritic growth of cortical neurons in rodents, highest expression of CamKIIα occurs later during dendritic maturation when the arbor is elaborated (Cline, 2001; Wong and Ghosh, 2002; Redmond and Ghosh, 2005). Another kinase pathway activated in response of neuronal activity is Ras-MAPK signaling (Konur and Ghosh, 2005). In rat hippocampal pyramidal neurons, persistent dual phosphorylation of ERK1/2 (MAPKs) in response to calcium influx and Ras signaling is important for local cytoskeletal effects and transcriptional changes that lead to dendritic remodeling (Wu et al., 2001). One key protein important for dendritic growth is MAP2 (microtubule associated protein 2), which is phosphorylated by MAPK kinases in response to neuronal activity (Vaillant et al., 2002). In rat cortical neurons, both CaM kinases and Ras/MAPK signaling can regulate gene transcription in response to neuronal activity. This is mediated by phosphorylation of transcription factors, cAMP response element binding protein (CREB), calcium-responsive transactivator (CREST), LMO4 and NeuroD2 (Redmond et al., 2002; Aizawa et al., 2004; Redmond and Ghosh, 2005; Kashani et al., 2006). Both CREB and CREST bind the transcriptional coactivator, CREB binding protein (CBP) on phosphorylation, although the mechanisms by which transcription regulates dendritic growth are not well-understood (Aizawa et al., 2004). Many cytoplasmic and secreted proteins whose expression increases after activity have been identified. Some of these have shown clear and essential function in modulating dendrite and spine morphology such as candidate plasticity gene-15 (cpg15) (Nedivi et al., 1998), Arc (Peebles et al., 2010), Homer (Sala et al., 2001), and brain-derived neurotrophic factor (BDNF) (Lom and Cohen-Cory, 1999). Other than kinases belonging to the CaM kinase and MAP kinase families, cyclin dependent kinase 5 (CDK5) kinase is translocated to the nucleus in an activity dependent manner in hippocampal neurons where it regulates the transcription of BDNF by phosphorylating the transcriptional repressor MeCP2 (Cheung et al., 2007). It is likely that a quantitative and comprehensive analysis of activity induced kinases might reveal yet unidentified pathways that regulate activity dependent dendritic development.

#### Kinase Signaling Mediate Dendritic Growth in Response to Neurotrophic Factors

Neurotrophins are secreted growth-promoting proteins that are essential for dendritic development both in peripheral and central nervous system (CNS). The four neutotrophins, nerve growth factor (NGF), brain derived growth factor (BDNF) and neurotrophins 3 and 4 (NT-3, NT-4) signal by binding and activating their receptors, which are members of the tropomyosin receptor kinases (Trk) and the structurally unrelated p75 neurotrophin receptor (Huang and Reichardt, 2001). Trk proteins are receptor tyrosine kinases that dimerize and transactivate on binding their respective neurotrophin ligand. In the mouse CNS, neurotrophins regulate the dendritic growth of pyramidal neurons in the developing neocortex (McAllister et al., 1995). Each of the four neurotrophins rapidly increases the length and complexity of dendrites of cortical pyramidal neurons when applied exogenously (McAllister et al., 1995). Experiments involving Trk “receptor bodies,” which are fusion proteins of the ligand-binding domains of each Trk receptor and the Fc domain of human IgG, clearly demonstrate that removing endogenous neurotrophins has dramatic consequences for the dendritic growth of pyramidal neurons in developing cortex (Shelton et al., 1995; McAllister et al., 1997). Consistent with the results of adding exogenous factors, endogenous BDNF is required for the growth and maintenance of dendritic arbors of layer 4 neurons whereas endogenous NT-3 is required for the growth and maintenance of dendritic arbors of layer 6 neurons. The distribution of receptors and secreted neurotrophin is exquisitely regulated. Not only do neurons in each cortical layer respond to subsets of neurotrophins, but also within a single cortical layer, each neurotrophin elicits a unique pattern of dendritic changes. Further, there are distinct changes in apical vs. basal dendrites evident at the individual neuron level (Horch and Katz, 2002). This precise level of neuronal and dendritic specificity suggests that neurotrophins do not simply enhance neuronal growth but, rather, act instructively to modulate particular patterns of dendritic arborization (McAllister, 2000). Another level of kinase regulation is added by Cdk5, which phosphorylates TrkB at the intracellular juxtamembrane region. In hippocampal cultures, reducing Cdk5 activity or expression of a TrkB mutant lacking the Cdk5 phosphorylation site abolishes BDNF triggered dendritic growth (Cheung et al., 2007). Thus, kinases act as both a signal detector (neurotrophin factors or calcium influx) and a biochemical switch (phosphorylation state) that turn on in response to stimuli and play critical roles in dendritic growth and remodeling (Figure 1A).

#### Kinase Signaling That Control Dendritic Growth, Tiling and Self-Avoidance

The phosphoinositide-3 kinase (PI3K)–Akt–mammalian target of rapamycin (mTOR) signaling pathway promotes dendritic growth and branching through upregulation of protein and lipid synthesis. The lipid kinase PI3K signals through AKT and is inhibited by phosphatase, *P*hosphatase and *Ten*sin homolog deleted on chromosome Ten (PTEN). In neurons, mTOR senses and integrates nutrient and growth factor availability through extracellular signals such as BDNF, insulin, insulin-like growth factor 1 (IGF1), vascular endothelial growth factor (VEGF), ciliary neurotrophic factor (CNTF) and glutamate, to mediate neuronal proliferation, dendritic growth and synaptic function (Lipton and Sahin, 2014). Constitutively active Ras, PI3K, and Akt that activate the mTOR kinase induce growth and elaboration of dendrites in cultured hippocampal neurons. In these neurons, this effect can be potently blocked by mTOR inhibitor rapamycin or activation of PTEN that counteracts the action of lipid kinase PI3K (Jaworski et al., 2005). Loss of upstream regulators of mTOR either TSC1/2 or lipid phosphatase PTEN, result in structural neuronal defects. PTEN null mutant mice exhibit dendritic hypertrophy and macrocephaly (Kwon et al., 2003; Lugo et al., 2014). Loss of TSC1/2 increased size of both neuronal somata and dendritic arbors (Tavazoie et al., 2005). Conversely, dendritic complexity is reduced by inhibition of PI3K, knockdown of mTOR, or its effector p70 ribosomal S6 kinase that upregulates protein synthesis. MTOR is composed of two complexes mTORC1 and mTORC2. These share the mTOR kinase but comprise other distinct proteins such as Raptor and Rictor, respectively, that make the two complexes functionally distinct (Hoeffer and Klann, 2010). Both mTORC1 and mTORC2 are integral to the proper formation of dendrites and their arbor. Knockdown of their essential components Raptor and Rictor in rat hippocampal and cortical neurons inhibits dendritic growth (Urbanska et al., 2012).

The conserved Hippo kinase signaling pathway plays an important role in dendritic growth, tiling and function (Jan and Jan, 2010). Hippo is a conserved serine/threonine kinase of the Ste family, that is important for dendritic growth in *Drosophila* peripheral sensory neurons (Emoto et al., 2006). Hippo kinase mediates dendritic arbor growth and maintenance through the action of downstream kinase Wts. The mammalian homolog of hippo are MST1/2 kinases, which phosphorylates and activates kinases involved in cytoskeletal modulation including Ndr1/2 (nuclear Dbf2-related) kinases and Lats1/2 (large tumor suppressor 1/2) kinases (Emoto, 2011). In *Drosophila* and *C. elegans* sensory neurons, Ndr kinase is required for dendrite branching where it likely promotes neurite outgrowth and branching through Rho family GTPase (Emoto, 2011). Mammalian Ndr1/2 kinases analogous to the roles of their fly homolog Trc, limit dendrite branching and length in hippocampal primary cultures and *in vivo* (Ultanir et al., 2012). The ability of Ndr kinases to limit dendritic branching is mediated by its direct substrate Aak1 kinase which is a downstream substrate implicated in regulation of intracellular trafficking (Ultanir et al., 2012). In hippocampal neuron cultures, Ndr2 kinase was shown to regulate dendritic growth through phosphorylation of integrins, its subsequent translocation to the neurite tips where it facilitate neurite extension (Rehberg et al., 2014). The Tao family of Ste kinases are regulators of Hippo kinases signaling (Poon et al., 2011; Huang et al., 2014). The mammalian Tao kinase, Taok2, is a serine-threonine kinase that specifically regulates the development of basal, but not apical, dendrites in cortical pyramidal neurons (de Anda et al., 2012). The secreted guidance molecule Semaphorin3a activates its receptor Neuropilin, which in turn binds Taok2 and regulates Jnk kinase signaling development of basal dendrites (de Anda et al., 2012). Taok2 associates with microtubules (Mitsopoulos et al., 2003), as well as mediates septin phosphorylation (Yadav et al., 2017). Mechanistically, how interaction of Taok2 with various cytoskeletal elements contributes to its role in dendritic development is not well-understood.

Elaboration of the dendritic arbor through growth and branching is fine-tuned by mechanisms of self-avoidance and tiling, which prevent redundancy and increase efficient use of receptive field (Figure 1B). Dendrites avoid overlapping with other dendrites of the same neuron in a process known as self-avoidance, well-studied in *Drosophila* (Grueber et al., 2003; Jan and Jan, 2010). Dendrite self-avoidance is important in order to prevent self-crossing of dendrites, clumping of dendrites as well as to maximize the receptive field. In Purkinje cells, the liver kinase B1 (Lkb1) is developmentally expressed in the dendrites. Depletion of Lkb1 in these neurons results in increased dendritic crossing (Kuwako and Okano, 2018). Exogenous expression of salt inducible kinase 1 (Sik1), a downstream target of Lkb1 kinase is able to rescue self-crossing defects through regulation of the guidance cue receptor Robo2 (Kuwako and Okano, 2018), these data suggest that the Lkb1-Sik1 kinase pathways is required for dendritic self-avoidance in cerebellar Purkinje neurons. Dendritic tiling ensures that dendrites of different neurons of the same type avoid each other (Grueber and Sagasti, 2010; Jan and Jan, 2010). Dendritic tiling has been best studied in the *Drosophila* peripheral sensory da neurons and in retinal ganglion cells. In flies, loss of either the serine/threonine kinase, Tricornered (Trc), or Furry (Fry), a protein required for Trc kinase activity, leads to sensory neurons that produce excessive numbers of dendritic branches, which fail to tile normally (Emoto et al., 2004).

### Regulation of Dendritic Spine and Synapse Development by Kinases

Dendritic spines are actin-rich protrusions on the dendritic membrane of most excitatory neurons. Early in development, thin actin filopodia extend from dendritic branches, which then mature into stable mushroom shaped spines likely on contact with the axonal membrane (Matus, 2000; Yuste and Bonhoeffer, 2004). However, spines retain remarkable structural plasticity even after development, and on exposure to the right stimuli can extensively retract, morph or become larger (Hering and Sheng, 2001). Spines are enriched in F-actin that contributes greatly to their structure and plasticity (Hotulainen and Hoogenraad, 2010). Repetitive firing of synapses, such as that which occurs during high-frequency synaptic stimulation of hippocampal neurons (long term potentiation), leads to an increase in F-actin which causes the spine to enlarge (Okamoto et al., 2004). Conversely, in long-term depression, decrease in F-actin/G-actin ratio causes dendritic spine shrinkage (Zhou et al., 2004). In addition to actin, other cytoskeletal elements such as septin and microtubules regulate dendritic spines structure. In cortical and hippocampal neurons, septin proteins mark the site of dendritic filopodia extension, and are important for dendritic spine stability (Xie et al., 2007; Ewers et al., 2014; Yadav et al., 2017). Interestingly, transient entry of microtubules into dendritic spines of hippocampal neurons regulates actin dynamics and spine morphology (Jaworski et al., 2009). It is therefore, not surprising, that several kinases (Figure 1C) and GTPases that regulate actin, septin and microtubule dynamics are implicated in spine formation and its plasticity (Hotulainen and Hoogenraad, 2010; Lin and Koleske, 2010).

Several kinases modulate spinogenesis through regulation of the actin cytoskeleton (Figure 1D). In addition to their role in dendrite development (described above) the calcium/calmodulin-dependent protein kinase II (CaMKII) is important for dendritic spine formation. In hippocampal neurons, the two main neuronal CaMKII isoforms have distinct roles, while CaMKIIα regulates synaptic strength, the CaMKIIβ isoform controls dendritic spine morphology and synapse number *via* its ability to bundle actin filaments (Fink et al., 2003; Okamoto et al., 2007). Knock-in mice expressing kinase dead CaMKIIα, show impaired learning and memory as well as a loss of long-term potentiation (Yamagata et al., 2009). Neuronal activity or NMDA receptor activation regulates spine morphogenesis by mediating Ca^2+^ influx into postsynaptic neurons, which modulate the activity of many actin binding proteins, including CaMKIIβ (Lisman et al., 2002). The dual specificity kinase Dyrk1a, negatively regulates filopodia and spine formation through phosphorylation of N-WASP, an actin filament assembly protein (Park et al., 2012). In cultured hippocampal neurons, N-WASP activates the actin branching protein Arp2/3, which is required for spine formation (Wegner et al., 2008). LIM-kinase1 (LIMK1) inhibits the activity of actin depolymerizing protein cofilin by phosphorylation (Yang et al., 1998) and hence affects dendritic spine morphology and synaptic function (Meng et al., 2002). LIM-Kinase1 (Limk1) functions as an actin destabilizer. Hippocampal neurons in Limk1 knockout mice exhibit aberrant spines and enhanced LTP (Meng et al., 2002). Another actin destabilizing kinase is PKC, several isoforms of which are enriched at the synapse. PKC phosphorylates myristoylated, alanine-rich C-kinase substrate (MARCKS) inhibiting its ability to cross link the actin cytoskeleton to membrane thereby destabilizing dendritic spines in hippocampal neurons (Calabrese and Halpain, 2005).

Receptor tyrosine kinases, notably Trk kinase and Eph/ephrin family members are important for dendritic spine formation. TrkB acts as a receptor for the neurotrophic factor BDNF and is crucial for neuronal plasticity such as structural remodeling associated with LTP (Huang et al., 2013). In rat hippocampal slices, BDNF stimulates activity of serine/threonine kinase p21 activated kinase (Pak), which inactivates cofilin through phosphorylation causing increase in spine size and stability. TrkB also activates Ras GTPase inducing spine enlargement and stability (Yasuda et al., 2006). Eph receptors expressed on dendrites are activated by ephrins on opposing membranes such as axonal/glial. Signaling through these receptors regulate dendritic spine and synapse formation or activity-induced LTP in hippocampal cultured neurons (Klein, 2009). Activation of Eph receptors leads to tyrosine phosphorylation of target molecules, such as proteoglycan syndecan-2, which clusters them at the postsynapse promoting spine maturation (Ethell et al., 2001). Activation of EphB on binding EphrinB leads to the receptor interaction with NMDA receptors and subsequent synaptic targeting of NMDA receptors (Dalva et al., 2000; Nolt et al., 2011). Further, Eph receptors also mediate structural changes in dendritic spines. Optogenetic local activation of expressed OptoEphb2 in dendrites led to rapid actin polymerization causing filopodial growth. While inhibition of Rac1 and Cdc42 did not abolish OptoEphB2-induced actin polymerization, Abelson tyrosine-protein kinase 2 (Abl2/Arg) were found to be downstream effector of filopodia growth in dendrites (Locke et al., 2018). Hippocampal neurons derived from EphB1/B2/B3 receptor triple knockout mice are unable to form mature dendritic spines (Henkemeyer et al., 2003), consistent with the essential role of Eph tyrosine kinases in spinogenesis. Another pathway that contributes to EphB2-mediated dendritic spine stabilization is FAK kinase that activates RhoA-ROCK-LIMK-1 pathway to suppress cofilin activity and remodel dendritic spines (Shi et al., 2009; Koleske, 2013).

In addition to actin, the septin cytoskeleton plays essential roles in dendritic spine morphogenesis (Figure 1D). Septin7 localizes to the base of dendritic spines at the spine neck and is required for spine formation (Tada et al., 2007; Xie et al., 2007) and spine stability (Ewers et al., 2014) in both hippocampal and cortical neurons. The serine/threonine kinase TAOK2 directly phosphorylates Septin7 at an evolutionarily conserved residue. In cultured hippocampal neurons, phosphorylation at its C-terminal tail promotes septin7 translocation from the base of the dendritic spine to spine head, where it associates and stabilizes the synaptic scaffold protein PSD95 (Yadav et al., 2017). TAOK2 depletion or expression of phospho-dead Septin7 leads to exuberant filopodial extension and inhibits spine maturation (Yadav et al., 2017).

### Aberrant Kinase Signaling Contributes to Dendritic Dysfunction in Neurological Disorders

Structural and functional dendritic defects are strongly associated with several neurodevelopmental and psychiatric disorders including autism spectrum disorder, schizophrenia, and Down syndrome (Raymond et al., 1996; Kulkarni and Firestein, 2012). Homeostatic balance between dendritic stability and instability is perturbed during neurodegenerative diseases or injury insults such as stroke. Most neurodegenerative diseases lead to dystrophy of dendrites and synapses, with some evidence suggesting that synaptic dysfunction precedes axonal degeneration (Gan et al., 2018). The evidence implicating kinase pathways in neurological disorders, and the potential mechanisms through which mutations in these kinase pathways contribute to disease are outlined below (Table 1).

**Table 1:** Kinase signaling pathways implicated in neurological diseases and their role in dendrite development.

**Kinase**	**Role in dendrite morphogenesis**	**Model system**	**Cell type/brain region**	**Manipulation**	**Phenotype**	**Disease association**	**References**
ERK1/2	Activity dependent growth through gene regulation, dendritic remodeling, phosphorylation of MAP2	Rat; Transgenic mouse	Hippocampal neurons; Sympathetic neurons	Pharmacological inhibition	Loss of filopodial stability in dendritic spine formation; loss of dendritic formation	16p11.2 CNV	Wu et al., 2001; Vaillant et al., 2002; Blizinsky et al., 2016
GSK3b	Neurite growth, specification of axons	Mouse, human tau transgenic mice; postmortem brain	Cortex, spinal cord	Pharmacological inhibition	Attenuated tau hyperphosphorylation	AD	Ferrer et al., 2002; Tackenberg et al., 2013; Griebel et al., 2019
FYN	Upstream of CDK5, regulation of cytoskeletal dynamics	Mouse	Hippocampal neurons	Pharmacological inhibition	Rescues synaptic depletion	AD	Rong et al., 2001; Ittner et al., 2010; Kaufman et al., 2015
DYRK1A	Regulates filopodia and spine formation through phosphorylation of N-WASP, Phosphorylates tubulin	Mouse; transgenic mouse	Dissociated mouse cortical and pyramidal neurons	Overexpression; pharmacological inhibition	Overexpression reduces dendritic arbor and spine density. Thinner spines. Inhibition leads to reduced tau hyperphosphorylation and aggregates.	AD, DS, ASD	Courcet et al., 2012; Martinez de Lagran et al., 2012; Park et al., 2012; Ori-McKenney et al., 2016; van Bon et al., 2016; Dang et al., 2018; Melchior et al., 2019
CaMKIIa	Stabilizes dendritic arbor, regulates synaptic strength	Mouse, *Xenopus*	Hippocampal neurons, optic tectal neurons	Catalytic domain mutation (E183V)	Increased dendritic arborization and decreased dendritic spine density.	ASD	Shen et al., 1998; Wu and Cline, 1998; Küry et al., 2017; Stephenson et al., 2017; Akita et al., 2018
CaMKIIb	Promotes actin polymerization increases filopodial extension, regulation of spine number and morphology through actin bundling	Rat	Hippocampus; hippocampal neurons	RNAi KD	Reduced number of mature dendritic spines	ASD	Shen et al., 1998; Fink et al., 2003; Okamoto et al., 2007; Küry et al., 2017; Akita et al., 2018
PI3K	Growth and elaboration of dendrites through Pi3k-Akt-mTOR axis, polarity	Rat	Hippocampal neurons	Pharmacological Inhibition	Reduced dendritic number and crossings	ASD, AD	Jaworski et al., 2005; Jansen et al., 2015; Winden et al., 2018
AKT	Growth and elaboration of dendrites through mTOR	Rat	Hippocampus	Constitutive activation	Increased arborization and increased spine size.	ASD	Jaworski et al., 2005; Jansen et al., 2015
mTOR	Growth and elaboration of dendrites	Mouse	Hippocampal neurons; Olfactory bulb neurons	RNA interference; Conditional knockout	Simplification of dendritic arbor	Cortex structural defects; ASD	Zhou et al., 2009; Urbanska et al., 2012; Mirzaa et al., 2016; Sato, 2016; Skalecka et al., 2016
PAK1/2	Regulation of spine size through inactivation of cofilin	Mouse; rat	Hippocampus	Knockout	Decreased number of immature dendritic spines	ASD, ID	Asrar et al., 2009; Harms et al., 2018; Horn et al., 2019; Kernohan et al., 2019
MET	Dendritic complexity, spine morphogenesis	Mouse, transgenic mouse	Hippocampal neurons	Overexpression; RNAi KD	Overexpression Increases dendritic spine density, dendritic intersections. RNAi reduces dendritic spine density and branches.	ASD	Campbell et al., 2007; Thanseem et al., 2010; Qiu et al., 2014
TAOK2	Basal dendrite development in cortical neurons, dendritic spine formation through septin 7	Mouse, rat	Cortical neurons; hippocampal neurons	RNAi KD, knockout	Reduced basal dendrite number and complexity; reduction in mature dendritic spines.	ASD, AD, 16p11.2 CNV	de Anda et al., 2012; Tavares et al., 2013; Yadav et al., 2017
CDKL5	Dendritic complexity	Mouse	Cortical neurons	Knockout	Reduction in spine density, spine stability and PSD95 puncta	CDKL5 Syndrome, ID	Weaving et al., 2004; Bahi-Buisson et al., 2012; Fuchs et al., 2014; Sala et al., 2016; Baltussen et al., 2018; Tang et al., 2019
LRRK2	Maintenance of dendritic length and branching	Mouse, *Drosophila*	Cortical neurons, slices, sensory neurons	Mutation G2019S	Mislocalized tau in dendrites leading to dendritic degeneration. Phosphorylated α-synuclein accumulation	PD	MacLeod et al., 2006; Lin et al., 2010; Di Maio et al., 2018
PINK1	Dendritic growth and arborization	Mouse	Primary cortical and midbrain dopaminergic neuron	Overexpression; knockout	Increased dendritic length on overexpression. Knockout causes dendritic length reduction.	PD	Valente et al., 2004; Dagda et al., 2014
ULK4	Pseudokinase, promotes microtubule acetylation; dendritic arborization	Mouse	Cortical neurons	Knockdown	Impaired neuritogenesis.	SZ	Lang et al., 2014, 2016; Liu et al., 2016

#### Autism Spectrum Disorder

Autism spectrum disorder (ASD) is a neurodevelopmental disorder with a strong genetic basis, and is defined by deficits in social communication, language development as well as repeated behaviors (Geschwind and Levitt, 2007). Several kinases have been genetically associated with autism spectrum disorders. *DYRK1A* has one of strongest genetic association with ASD (De Rubeis et al., 2014; Iossifov et al., 2014). The encoded protein DYRK1a is a dual-specificity tyrosine phosphorylation-regulated serine/threonine kinase. Several ASD associated variants of *DYRK1A* affect its kinase function causing either loss or gain of kinase activity. Mouse hippocampal neurons transfected with these variants show defects in neuronal development including in dendritic outgrowth and dendritic spine density (Dang et al., 2018). Further overexpression of these pathogenic variants in developing mice embryos perturb neuronal migration *in vivo* (Dang et al., 2018). DYRK1A mutations lead to syndromic form of autism and intellectual disability with many shared features. *DYRK1A* is affected in 21q22 microdeletion in human, and is associated with growth retardation, primary microcephaly, facial dysmorphism, seizures, ataxic gait, absent speech and intellectual disability (Møller et al., 2008; Courcet et al., 2012; Ji et al., 2015; van Bon et al., 2016).

Aberrant signaling through the PI3K/AKT/mTOR pathway is associated with ASD (Winden et al., 2018). A range of structural brain abnormalities are associated with mutations in the mTOR pathway. Activating mutations in PI3K/AKT/mTOR pathway result in megalencephalies and hemimegalencephalies associated with ASD (Jansen et al., 2015). Mutations in *PTEN* are linked to macrocephaly and ASD (Butler et al., 2005). Pten knockout mice exhibit enlarged dendritic arbors and neuronal soma and exhibit autism-like behavior (Lugo et al., 2014). Mutations in genes involved in the mTOR signaling pathway have been identified in some cases of syndromic ASD (Mirzaa et al., 2016). While mutations that inhibit mTOR are associated with microcephaly, hyperactive mTOR signaling is associated with monogenic ASD. Inhibition of mTOR signaling is a potential pharmacotherapy for ASD (Sato, 2016).

*TAOK2* is an autism susceptibility gene encoding a serine/threonine kinase. *De novo* mutations in *TAOK2* have been found in ASD patients (Richter et al., 2018). TAOK2 is one of the two kinases within the 16p11.2 gene locus, a region prone to copy number variations (CNV) associated with ASD and schizophrenia (Kumar et al., 2008; Weiss et al., 2008; McCarthy et al., 2009). ASD patients with 16p11.2 CNV exhibit a strong correlation between *TAOK2* expression and head circumference (Luo et al., 2012). Further, Taok2 knockout mice exhibit increased total brain volume compared to wildtype (Richter et al., 2018), suggesting that perturbation in *TAOK2* gene dosage might contribute to reciprocal brain size difference associated with the 16p11.2 CNV. However, mechanisms of whether and how TAOK2 might affect brain size have not been elucidated. TAOK2 kinase regulates the microtubule cytoskeleton (Moore et al., 2000; Mitsopoulos et al., 2003), septin cytoskeleton (Yadav et al., 2017) and RhoA signaling (de Anda et al., 2012). It is likely that many of these signaling pathways converge to mediate structural changes in the brain. In cultured hippocampal neurons, depletion or expression of kinase dead TAOK2 results in a loss of mature mushroom-shaped spines (Ultanir et al., 2014; Yadav et al., 2017; Richter et al., 2018), increased shaft synapses (Yadav et al., 2017). Further in cortical neurons, TAOK2 is required for proper basal dendrite development (de Anda et al., 2012). Interestingly, *de novo* mutations in another member of the TAO family, TAOK1, are associated with neurodevelopmental delay (Dulovic-Mahlow et al., 2019).

Additional kinases associated with ASD are MET, CaMKII and the PAK kinases. *MET* is associated with ASD and encodes a receptor tyrosine kinase MET (Campbell et al., 2007; Thanseem et al., 2010). Loss or gain of function of Met leads to opposing changes in dendritic complexity, spine morphogenesis, and timing of glutamatergic synapse maturation in CA1 hippocampal neurons (Qiu et al., 2014). *De novo* mutations in *CAMK2A* and *CAMK2B* are associated with autism, intellectual disability and neurodevelopmental disorders (Küry et al., 2017; Akita et al., 2018; Chiocchetti et al., 2018). *De novo* mutation in the CaMKIIα catalytic domain (E183V) was identified in a ASD proband (Stephenson et al., 2017). In cultured hippocampal neurons, the E183V mutation reduces CaMKIIα targeting to dendritic spines, increases dendritic arborization and decreases dendritic spine density. Mice with a knock-in CaMKIIα-E183V mutation display aberrant behavioral phenotypes, including hyperactivity, social interaction deficits, and increased repetitive behavior (Stephenson et al., 2017). Characterization of 19 rare *de novo* CAMK2A or CAMK2B variants identified in individuals with intellectual disability revealed that mutations that decreased or increased CAMKII autophosphorylation at Thr286/Thr287 also affected neuronal migration (Küry et al., 2017). PAK1 and PAK2 kinases are associated with autism and neurodevelopmental delay (Harms et al., 2018; Horn et al., 2019; Kernohan et al., 2019). *PAK1* mutations in patients with intellectual disability are located within or proximal to the autoinhibitory switch domain, suggesting a gain of function mechanism of disease (Harms et al., 2018). The Pak1/2 serine/threonine protein kinases regulate cell motility, cell cycle progression, apoptosis or proliferation through downstream GTPases Cdc42 and Rac1. PAK2 is encoded in the 3q29 genomic region, deletion of which can result in numerous neurodevelopmental defects including ASD (Quintero-Rivera et al., 2010). Haploinsufficiency of *PAK2* in mice results in decreased spine density and synapse number in the hippocampus (Wang et al., 2018b). In addition to PAK1/2, mutations in *PAK3* gene are associated with X-linked intellectual disability (Allen et al., 1998).

#### CDKL5 Syndrome

CDKL5 Syndrome is a rare X-linked genetic disorder that results in severe neurodevelopmental impairment, infantile seizures and intellectual disability (Weaving et al., 2004). Mutations in *CDKL5* gene encoding a serine/threonine kinase is causative of the syndrome. The severity of the disease seems to depend on the site of mutation, where those in the kinase domain are more pathogenic (Bahi-Buisson et al., 2012). CDKL5 knockout mouse exhibit dendritic hypotrophy in granule cells of the hippocampus (Fuchs et al., 2014). Enhanced NMDAR signaling and circuit hyperexcitability were shown to underlie autistic-like features in mouse models of CDKL5 Syndrome (Tang et al., 2019). Recently, direct target substrates of CDKL5 in the brain were identified, which included microtubule regulators Microtubule Associated Protein 1S (MAP1S), Microtubule End Binding Protein 2 (EB2) and RhoGTPase activator ARHGEF2 (Baltussen et al., 2018). Importantly, hypophosphorylation of these targets were confirmed *in vivo* as well as in induced pluripotent stem cell (iPSC) derived neurons differentiated from CDKL5 patients, suggesting that these are physiologically relevant kinase and substrates. Further, this important study also identified the consensus sequence of CDKL5 phosphorylation as RPXpS motif (Baltussen et al., 2018), which will be useful for identification of other downstream substrates.

#### Schizophrenia

*ULK4* is a rare susceptibility gene for schizophrenia (Lang et al., 2014; Tassano et al., 2018), a devastating neuropsychiatric disease with high heritability but few monogenetic associations. The ULK (UNC51-like) kinase family member ULK4 is classified as a pseudokinase, as it is catalytically inactive. While ULK can bind ATP molecules it does not have phosphotransfer activity (Khamrui et al., 2020). The expression of *ULK4* is neuron-specific and developmentally regulated, and its depletion in mice leads to defects in neural proliferation, migration as well as reduced dendritic arborization of cortical neurons (Lang et al., 2016). Knockdown of Ulk4 disrupts the composition of microtubules by reducing tubulin acetylation. Targeted disruption of the Ulk4 in the cortex decreases the neural stem cell pool at birth, which significantly reduced cerebral cortex size in postnatal mice (Liu et al., 2016). The implications of these changes in pathogenesis of schizophrenia are unknown.

#### 16p11.2 CNV Syndrome

Copy number variation (CNV) in the 16p11.2 genomic locus are strongly associated with neurodevelopmental disorders including ASD, schizophrenia, and structural brain changes (Kumar et al., 2008; Weiss et al., 2008; McCarthy et al., 2009; Qureshi et al., 2014; Owen et al., 2018). This genomic locus spans 29 annotated genes, two of which encode kinases: *TAOK2* and *MAPK3*. Transcriptomic analysis of 16p11.2 CNV patients suggests there is a strong correlation between *TAOK2* gene expression and head circumference (Luo et al., 2012). Further, TAOK2 knockout mice exhibit increased total brain volume compared to wildtype (Richter et al., 2018). Dissociated primary cortical neurons from 16p11.2 microduplication mice model show increased dendritic elaboration, which was rescued with ERK1 inhibitors (Blizinsky et al., 2016), suggesting increase in MAPK3 dosage might contribute to changes in brain structure and function. Paradoxically, ERK1 inhibition in 16p11.2 deletion also seems to rescue cortical defects in mice models. It is important to note that mouse models of 16p11.2 CNVs do not faithfully recapitulate the human condition in terms of structural brain changes (Portmann et al., 2014; Deshpande et al., 2017), and human relevant models of this CNV are needed to understand the mechanisms of many defects associated with these CNVs. Interestingly, *TAOK2* and *MAPK3*, are the only two genes in this locus that have been independently associated with autism (Pinto et al., 2014; Richter et al., 2018).

##### Down Syndrome

Down Syndrome (DS) is a severe neurodevelopmental disorder caused by presence of an extra copy (or parts) of chromosome 21 (Dierssen, 2012). Postmortem analysis of neuronal morphology in DS patients showed increased complexity of dendritic arbors in early postnatal period followed by much reduced dendritic length and arborization in older children (Becker et al., 1986). Among the DS Critical genes, increased dosage of *DYRK1A* has been identified to play a crucial role in the disease pathology. Dyrk1a overexpression in mouse neocortex inhibits neural stem cell proliferation and leads to premature neuronal differentiation (Yabut et al., 2010). Overexpression of Dyrk1A leads to reduced dendritic arbor complexity and synaptogenesis in layer II/III pyramidal cells, indicating this kinase is a major contributor to the dendritic phenotypes in DS (Martinez de Lagran et al., 2012). Both the mammalian Dyrk1a and its *Drosophila* ortholog minibrain regulate dendritic morphogenesis through direct phosphorylation of β-tubulin which inhibits microtubule polymerization (Ori-McKenney et al., 2016).

##### Alzheimer's Disease

Alzheimer's disease (AD) is a progressive neurodegenerative disease that affects wide areas of the cerebral cortex and hippocampus. Human neuropathology data from AD patients suggests that dendritic abnormalities in AD are widespread and often are present in the early stages of disease. Dendritic abnormalities associated with AD include dystrophic dendrites, reduction in dendrite complexity, and loss of dendritic spines (Cochran et al., 2014). Hallmark features of AD include accumulation of extracellular insoluble forms of amyloid-β (Aβ) and intracellular aggregation of hyperphosphorylated microtubule associated protein tau in neurofibrillary tangles (Giacobini and Gold, 2013; Congdon and Sigurdsson, 2018). Aβ refers to peptides that are 38–43 amino acids in length, derived by the proteolytic cleavage of amyloid precursor protein (APP). Aβ is released into the extracellular matrix in oligomeric form where it is normally cleared by macrophages and microglia. Defects in efficient clearing of Aβ and certain oligomeric Aβ conformations lead to formation of fibrils that ultimately form amyloid plaques (Ittner and Ittner, 2018). Plaques induce neurotoxicity through numerous pathways, including through recruitment of the microtubule binding proteins tau (Masters et al., 2015). Experiments in hippocampal neurons show that Aβ oligomers accumulate at synapses, inducing clustering and dysfunction of metabotropic glutamate receptors (Renner et al., 2010). Exposure to Aβ oligomers disrupts polarized trafficking causing mis-sorting of axonal proteins including tau into somatodendritic compartment. Defects in axonal trafficking of tau promotes axonal degeneration and corresponding decrease in synaptic inputs (Zempel et al., 2010). Further, missorted tau in dendrites induces tubulin polyglutamylation which recruits the microtubule severing protein spastin, ultimately leading to dendritic dystrophy (Zempel et al., 2013). Of note, disease modifying treatments targeting Aβ, have so far failed in clinical trials. Recent evidence suggests that amyloid deposition is not strongly correlated with cognition in multivariate analyses. Hyperphosphorylated tau, as well as synaptic and neuronal loss are, however, associated with memory deficits (Giacobini and Gold, 2013). AD is associated with high amount of hyperphosphorylated tau. Tau contains 77 potential serine/threonine and 4 tyrosine phosphorylation sites clustered in the proline-rich region and the tail domain adjacent to the microtubule targeting domains (Noble et al., 2013) Tau hyperphosphorylation decreases its binding to microtubules. As tau becomes progressively hyperphosphorylated, deficits in molecular chaperones and degradation contribute to tau oligomerization and paired helical filament formation, ultimately forming neurofibrillary tangles (Iqbal et al., 2016; Ittner and Ittner, 2018). Emergent evidence suggests that AD is a synaptopathy (Li et al., 2018). Synaptic dysfunction due to pathogenic Aβ oligomers and tau pathology is one of the earliest signs of disease, preceding synaptic loss and neurodegeneration (Selkoe, 2002; Hoover et al., 2010; DeVos et al., 2018). In addition to its axonal role, tau plays a dendritic function important for postsynaptic targeting of Fyn kinase, a modulator of NMDA receptor activity. Tau KO mice exhibit disrupted postsynaptic targeting of Fyn, which reverses the excitotoxicity caused by NMDA receptor dysfunction due to Aβ toxicity (Ittner et al., 2010). In addition to Fyn kinase, dysfunction in several kinase pathways have been implicated in AD. Overactivation of Gsk3β (Lauretti et al., 2020) and Dyrk1a (Coutadeur et al., 2015) has been independently shown to increase tau phosphorylation as well as Aβ production. Overactivated TAOK2 kinase was found in the neurofibrillary tangles in AD postmortem brain (Tavares et al., 2013), and its inhibition reduces tau phosphorylation in cellular models (Giacomini et al., 2018). Another important kinase pathway that has emerged in AD is the Cdk5. Deregulation of Cdk5 by overexpression of its activator p25 triggers progressive neurodegeneration and neurofibrillary tangle formation in mice (Cruz and Tsai, 2004). In addition to its effect on microtubule stability and synaptic function, hyperphosphorylated tau promotes Aβ toxicity mediated neuropathology (Ittner et al., 2010; Mairet-Coello et al., 2013), hence targeting tau hyperphosphorylation might prove to be a viable therapeutic strategy for AD. Several small molecule inhibitors targeting kinases that phosphorylate tau are currently in clinical trial for AD (Table 2) (Tell and Hilgeroth, 2013; Krahn et al., 2020).

**Table 2:** Kinase inhibitors in clinical trials for the treatment of neurological disorders.

**Kinase**	**Disease**	**Agent**	**Mechanism of action**	**Therapeutic goal**	**Clinical trial ID**
GSK3β	AD	Tideglusib	Non-ATP competitive inhibitor of GSK3β	Reduction of tau phosphorylation	NCT00948259 NCT01350362 NCT02586935 (USA)
DYRK1A	AD DS	SM07883 Epigallocatechin-3-gallate (EGCG)	ATP competitive inhibitor of DYRK1A Non-ATP competitive inhibitor of DYRK1A	Inhibition of tau hyperphosphorylation, aggregation, and NFT formation Amelioration of DS cognitive symptoms through DYRK1A inhibition	ACTRN12619000327189 (Australia) NCT01394796 (USA)
FYN	AD	Saracatinib	ATP-competitive inhibitor of Src family of tyrosine kinases	Reduced Fyn activation by Aβ	NCT02167256 (USA)
ABL	AD	Nilotinib	ATP competitive inhibitor of ABL	Stabilization of levels of phosphorylated tau, total tau and Aβ	NCT02947893 (USA)
p38 MAPK	AD DLB	Neflamapiod	ATP-competitive inhibitor of p38 MAPK	Decrease neuroinflammatory markers, reduced decline in cognitive function	NCT03402659 NCT03435861 NCT04001517 (USA)
LRRK2	PD	DNL151	Inhibition of LRRK2 kinase	Decrease neuroinflammatory markers	NCT04056689 (USA)

##### Parkinson's Disease

Parkinson's disease (PD) is pathologically defined by the neurodegeneration of dopaminergic neurons of the substantia nigra, and is characterized by the presence of cytoplasmic inclusions composed of α-synuclein protein aggregates called Lewy bodies (Poewe et al., 2017). Some of the strongest disease associated genes in Parkinson's disease encode for kinases; LRRK2 and PINK1. Mutations in leucine-rich repeat kinase 2 (LRRK2) underlie an autosomal-dominant, inherited form of PD. The PD-associated LRRK2 mutations display disinhibited kinase activity and induce a progressive reduction in dendrite length and branching in primary cortical cultures and *in vivo* mouse models (MacLeod et al., 2006). Increased LRRK2 kinase activity was observed in idiopathic PD, and in neurons exposed to mitochondrial toxins, suggesting that LRRK2 kinase activity might have a broader role in PD pathogenesis (Di Maio et al., 2018). Small-molecule LRRK2 kinase inhibitors have shown promise in preclinical models of PD, and has brought LRRK2 to the forefront of disease modifying efforts in PD (Tolosa et al., 2020). Homozygous loss of function mutations in PTEN-induced kinase 1 (PINK1) are associated with early onset PD (Valente et al., 2004). PINK1 encodes a serine/threonine kinase that acts as a sensor for mitochondrial health. In healthy mitochondria, PINK1 is targeted to mitochondria where it is rapidly degraded. In unhealthy membrane potential (ΔΨm)-deficient mitochondria, however, PINK1 accumulates and recruits an E3-ubiquitin ligase Parkin, which in turn initiates mitophagy (Okatsu et al., 2012). Loss of function PD associated PINK1 mutations perturb normal neuronal mitophagy, and accumulated damaged mitochondria in neurons lead to disease pathology. In mouse primary cortical and midbrain dopaminergic neurons, PINK1 kinase activity was found to promote dendritic arborization and its depletion resulted in shortened dendritic length (Dagda et al., 2014). While mechanisms through which PINK1 might regulate dendritic length are not well elucidated, there is some indication that control of mitochondrial motility and trafficking within dendrites by PINK1 could contribute to its dendritic role (Banerjee et al., 2017).

### Technologies to Dissect Kinase Signaling in Dendritic Structure and Function

Kinase signaling occurs in a spatiotemporally precise fashion, however, traditional biochemical tools do not provide information on when and where kinase activation occurs during neuronal development. A major challenge in understanding kinase function is the identification of direct substrates of the kinase of interest (KOI).

#### Kinase Sensors and Optokinases

**Figure 2:**
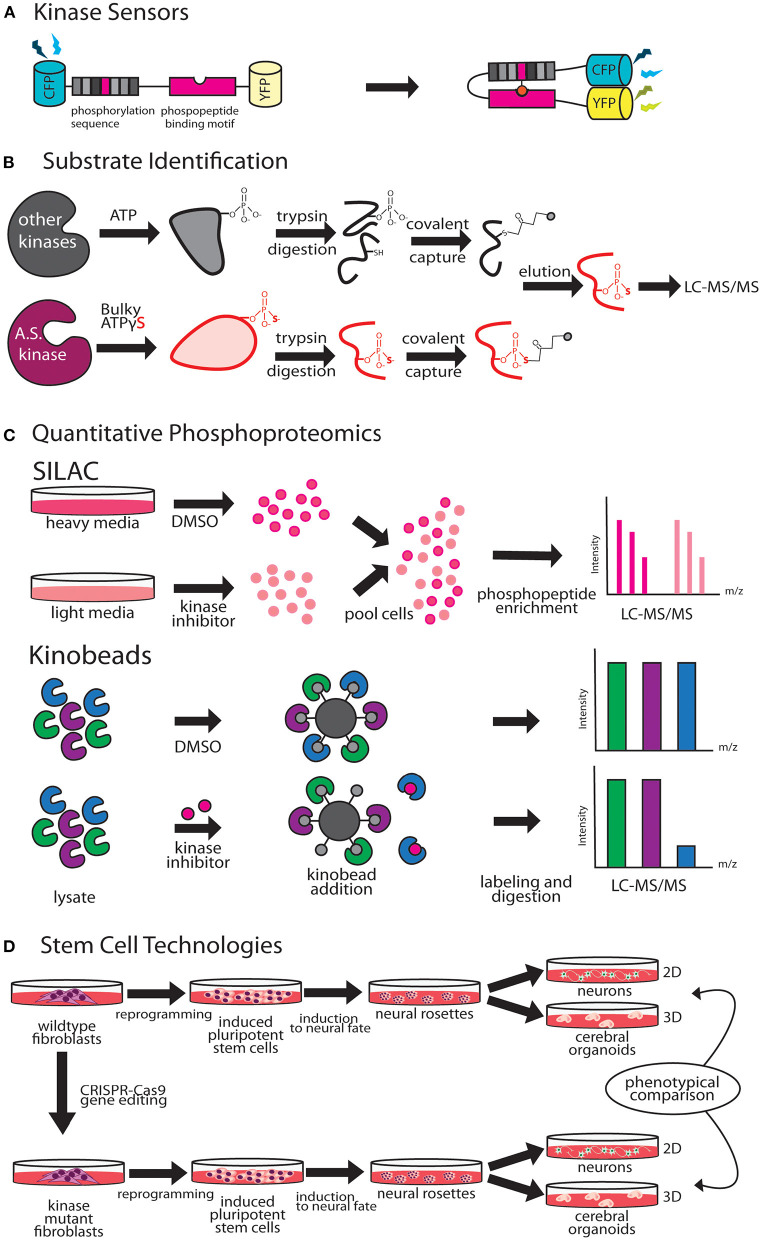
Emergent technologies to investigate kinase pathways. **(A)** FRET-based kinase sensors are comprised of kinase specific phosphopeptide sequence and a phosphopeptide binding motif. **(B)** Analog-sensitive kinases are an important tool for direct substrate identification through covalent capture of phosphopeptides and mass spectrometry. **(C)** Stable isotope labeling of amino acids in culture (SILAC), is a versatile metabolic labeling proteomic technique that can be applied to study the effect of kinase inhibitors on neuronal growth by quantifying changes in proteome. Kino-beads can be employed for profiling the neuronal kinome in healthy and disease states. **(D)** Human iPSCs have been used to model neurological diseases associated with kinase genes.

A key method for studying temporal and spatial kinetics of kinase signaling is the use of kinase sensors (Figure 2A). Fundamentally, kinase sensors are comprised of two parts, a sensing unit, which is sensitive to a phosphorylation event on substrate and a reporting unit to indicate the phosphorylation state (Oldach and Zhang, 2014). Fluorescent kinase sensors work in three ways (Turk, 2005). FRET kinase sensors rely on Förster resonance energy transfer (FRET) between a fluorophore donor attached to a peptide designed to harbor the kinase specific phosphorylation site, and an acceptor fluorophore bound phospho-peptide binding motif (Sato et al., 2002). The phosphopeptide sensing motif interacts with the phosphorylated peptide, bringing donor and acceptor fluorophores together achieving resonant fluorescence that can be visualized through microscopy. Environmentally sensitive kinase sensors utilize a phosphorylation sequence peptide conjugated to a fluorophore that shifts wavelengths or intensity when in close proximity to phosphate (Yeh et al., 2002; Sharma et al., 2008). Chelation sensitive sensors use fluorophores sensitive to Mg^2+^ concentrations found in the ATP binding site of the kinase, paired with a phosphopeptide (Shults and Imperiali, 2003). Activation of ERK, PKA, and CaMKIIα in single dendritic spines during LTP has been studied using FRET based kinase sensors (Tang and Yasuda, 2017). Optogenetic regulation of kinases enables fast, reversible, and non-invasive manipulation of protein kinase activities providing exquisite control on regulation by bypassing upstream factors like growth factors, protein kinase inhibitors or chemical crosslinker that induce changes in kinase activity (Leopold et al., 2018). Design of opto-kinases is based on the plant photoreceptor domain light, oxygen, or voltage (LOV) or photoswitchable caging using Dronpa fluorophore. The light, oxygen, or voltage (LOV) domains are the sites for initial photochemistry in blue light photoreceptors in plant flavoprotein kinases, which have inspired creation of light activated kinases (Crosson and Moffat, 2002). LOV domains can be used to drive light mediated homodimerization of engineered tyrosine kinase domain thereby leading to their activation. The dimeric protein, pdDronpa, dissociates in cyan light and reassembles in violet light (Zhou et al., 2017). Switchable kinases have been designed by attaching two pdDronpa domains in the kinase domain thereby caging the kinase. When Dronpa dimers dissociate in cyan light it allows the kinase domains to come together to get activated, which can be rapidly shut down with violet light. Dronpa based photo-switchable (ps) psRaf1, psMEK1, psMEK2, and psCDK5 kinases were recently designed to uncover a direct and rapid inhibitory feedback loop from ERK to MEK1. Dronpa-kinases were also shown to work *in vivo* where they could acutely regulate synaptic vesicle transport (Zhou et al., 2017). Challenges such as low dynamic range, low signal to noise ratio, applicability to *in vivo* studies and control of expression level are some of the difficulties in design of useful kinase sensors (Oldach and Zhang, 2014). Iterative sensor optimization combined with improvements in imaging capabilities will further expand the scope of kinase sensors in understanding dendritic development and function.

#### Kinase Substrate Identification

Cascades of protein phosphorylation downstream of kinase activation make precise identification of direct substrates difficult. A breakthrough in this field was the development of a technique that enables covalent capture of analog-sensitive kinase substrates (Figure 2B). This technique involves genetic engineering of the KOI to allow for utilization of bulkier ATPγS analogs. Thiophosphorylated proteins that represent direct substrates of KOI are covalently captured by thiol-reactive iodoacetyl agarose beads, and identified by mass spectrometry (Blethrow et al., 2008). As this method also yields the site of phosphorylation on the identified substrate, validation of substrate identity can be easily performed by point mutation of these sites in downstream biochemical assays. This method has been used for identification of direct targets of several kinases that play key role in dendrite morphogenesis including Cdkl5 (Baltussen et al., 2018), Taok2 (Yadav et al., 2017), and Hippo kinase members (Ultanir et al., 2012, 2014). Biological function of KOI and validated substrates can then be inferred through independent methods such as purified *in vitro* kinase assays and rescue of biological phenotypes with phosphomimetic substrates. An innovative application of this method was the finding that certain kinases such as PINK1, can utilize artificial ATP analogs (kinetin triphosphate), more efficiently than ATP, enhancing kinase activity of wild type as well as rescuing effects of low activity PD associated PINK1 mutant (Hertz et al., 2013). A limitation of this technique is that since phosphorylation reaction is performed *in vitro* in a cell or tissue lysate, the physiological context of kinase and substrate localization is lost. This can lead to false positive substrates, and therefore further validation of candidate substrates identified through this technique is essential.

#### SILAC

Stable isotope labeling by amino acids in cell culture (SILAC) is a metabolic labeling technique that can be used to incorporate amino acids carrying specific heavy and light isotopes of C and N, allowing for simultaneous identification and quantitation of complex protein mixtures (Ong et al., 2002). SILAC can be employed in neuronal cultures or *in vivo* animal models to detect changes in the proteomic landscape upon different genetic or pharmacological perturbations (Krüger et al., 2008; Spellman et al., 2008). Therefore, SILAC can be a powerful method to study kinase pathways during dendrite development or disease (Figure 2C). SILAC was utilized to identify novel interactors of Tao kinases on phosphorylation by upstream kinase Mst3, to reveal unique interaction with cytoskeletal motor protein MyosinVa (Ultanir et al., 2014). Fragile X Syndrome is caused by transcriptional silencing of *Fmr1*, which encodes a protein that regulates mRNA translation in neuronal dendrites. SILAC labeling revealed profound up- or down- regulation of proteins related to synaptic structure and morphogenesis, dendritic mRNA transport, and synaptic transmission in Fmr1 knockout mouse cortical synapses compared to wild type (Liao et al., 2008). Improvements in SILAC technologies include use of “spike in” SILAC that can be utilized to compare more than three distinct comparisons (Geiger et al., 2010). However, optimization of proper spike-in-standard can be time consuming and variable based on the sample type (Wang et al., 2018a).

#### Quantitative Phosphoproteomics

Quantitative phosphoproteomics has emerged as a powerful tool to perform unbiased and quantitative measurement of changes in signaling pathways in normal and diseased states (Hosp and Mann, 2017). Chemical labeling methods like tandem mass tag (TMT) or iTRAQ allow for multiplexing and are powerful when used with human tissue samples as labeling is performed *in vitro* after obtaining the tissue lysate (Glibert et al., 2015; Navarrete-Perea et al., 2018). Dendritic differentiation and maturation occur in distinct stages, and proteomic techniques can be applied to systematically delineate kinase pathways that mediate each of these processes. The developmental proteomic profile of cultured rat hippocampal neurons at different stages was recently mapped. Here, a combination of stable isotope labeling and high-resolution liquid chromatography-tandem mass spectrometry (LC-MS/MS) was utilized to detect extensive remodeling of the neuronal proteome, where one third of 4,500 proteins quantified were found to undergo 2-fold change in expression during neuronal differentiation (Frese et al., 2017). Phosphoproteomic techniques are especially powerful in identification of signaling aberrations in disease states. Haploinsufficiency of the gene *SHANK3* causes Phelan-McDermid syndrome (PMDS) that is associated with a high risk of autism (Mitz et al., 2018). Unbiased, quantitative proteomics revealed profound changes in the phosphoproteome of Shank3-deficient neurons, including downregulation of Akt-mTORC1 signaling due to increased steady-state levels of upstream kinase, Cdc2-like kinase 2 (Clk2) (Bidinosti et al., 2016). Pharmacological and genetic activation of Akt or inhibition of Clk2 relieved synaptic and behavioral deficits in PMDS patient-derived neurons and mouse models, thereby highlighting the value of using unbiased proteomic approaches in discovery of novel drug targets. Another powerful example is the use of phosphoproteomics screening analysis of Parkinson disease associated kinase LRRK2, in combination with different pharmacological inhibitors that uncovered Rab GTPases as key LRRK2 substrates, and pointed toward a new disease mechanism in PD (Steger et al., 2016).

#### Kinome Profiling

Immobilized broad-spectrum kinase inhibitors can be used in affinity pulldown to probe full-length kinases from whole neuronal or brain tissue proteomes (Bantscheff et al., 2007). This chemical proteomics method uses as multiplexed kinase inhibitor beads or kinobead-profiling enabling simultaneous profiling of over 200 kinases in a single experiment. Combining kinobead pulldown with SILAC or isobaric chemical labeling can increase analytical throughput dramatically, as well as allow comparison of proteomes from normal and disease states (Golkowski et al., 2017). In addition, kinobeads can be used to map drug-induced changes in the phosphorylation state of the kinome, enabling analysis of signaling downstream of target kinases (Golkowski et al., 2020). A limitation of kinobeads is that their applicability in providing spatial and temporal information on the kinome has not been proven. Most applications of kinobeads to date have focused on whole cell or tissue samples to query the kinome. ATP probes can be used in a similar fashion for profiling the entire kinome, kinases and other ATP utilizing proteins such as ATPases. This complementary chemical proteomic method trademarked as KiNativ, utilizes highly reactive biotinylated acyl phosphate derivatives of ATP as an affinity tag to profile the cellular kinome (Patricelli et al., 2007). In addition, the KiNativ platform can be used to determine the proteomic response to specific kinase inhibitors, which allows characterization of inhibitor interactions with endogenous kinases in native conditions (Patricelli et al., 2011). Application of these advanced kinome profiling techniques to better understand and delineate kinase signaling in dendritic development and disease states would likely reveal novel mechanistic insight (Figure 2C).

#### Modeling Dendritic Dysfunction Using iPSC Derived Neurons

Human induced pluripotent stem cell (hiPSC) derived neurons can be a powerful tool to model dendritic development and for studying how kinase signaling contributes to development and dendritic dysfunction. Stem cells can be reprogrammed from affected patient fibroblasts or from health controls, and differentiated into different neuronal and non-neuronal cell types to model neurological diseases *in vitro* (Dolmetsch and Geschwind, 2011; Paşca et al., 2014). There are three primary applications of this technology in studying dendrite development. First, iPSCs from healthy individuals can be gene edited using CRISPR-Cas9 to introduce truncations and single nucleotide polymorphisms (SNPs) to model the effect of kinase disease variants (Vermilyea et al., 2020). Secondly, iPSCs derived from patients with known neurological disorder can be differentiated to study neuronal development (Figure 2D). Forebrain cortical neurons differentiated from iPSCs derived from fibroblasts of 16p11.2 deletion and duplication carriers, exhibit opposing changes in dendritic arbor size (Deshpande et al., 2017), suggesting that reciprocal changes in neuronal size could contribute to brain size defects associated with 16p11.2 CNVs. Neurons derived from iPS cells are an excellent human cellular model for investigating neuronal development in an isogenic background. For example, female iPSCs harboring *CDKL5*-mutations were shown to maintain X-chromosome inactivation, with clones expressing either mutant *CDKL5* allele or the wild-type allele (Amenduni et al., 2011). Neurons differentiated from patient iPSCs with *CDKL5* mutations develop increased number of dendritic protrusions (Ricciardi et al., 2012). Another study utilized iPSC derived neurons from patients with distinct mutations to show that loss of CDKL5 led to a decrease in phosphorylation of CDKL5 substrate EB2. A microtubule regulator, EB2 phosphorylation status was shown to be critical for microtubule dynamics within dendrites (Baltussen et al., 2018). Finally, human iPSC derived 2D neuronal and 3D organoid models can be used in high throughput screens, for identification of novel modulators of dendrite development. A recent high-throughput screening of human iPSC-derived neurons focused on neurite growth employed a collection of 4,421 bioactive compounds, and identified 108 hit compounds, including 37 approved drugs, that regulate neurite growth (Sherman and Bang, 2018). Human iPSC derived neurons can be used as a preclinical model for drug toxicity tests relevant to human physiology, an issue that has hampered progress in generation of new therapeutics (Inoue et al., 2014). Continued innovation in the field of stem cell technologies have greatly improved reproducibility and reliability of iPS derived neurons. The prolonged developmental timeline of human neuronal development compared to rodents (Dolmetsch and Geschwind, 2011), however, necessitates further improvement that enable longer *in vitro* 2D and 3D culturing capacities, and allow for co-culturing of various brain cell types to recapitulate *in vivo* dendritic development, maturation and synaptic pruning (Kelava and Lancaster, 2016).

## Summary

Kinase signaling pathways act in a concerted fashion to mediate almost all aspects of dendritic development. These complex signaling pathways are elegantly intertwined, with key kinase signaling elements recurring throughout neuronal development. The human genome encodes a total of 518 kinases (Manning et al., 2002). While genetic models and GWAS analyses have identified several kinase-encoding genes implicated in neurological disease (Krahn et al., 2020), there remains much to discover about kinase function in dendrite development and how their dysregulation contributes to neuronal disease. Unbiased mapping of kinase signaling that instruct distinct stages of dendritic growth may reveal novel pathways that can be further genetically dissected. Comparative phosphoproteomic analyses of normal and disease states will serve as a powerful tool for future identification of novel therapeutic kinase targets. Advances in neurobiological and proteomic techniques will greatly facilitate the exploration of kinase pathways that impact dendrite structure and dysfunction in disease states.

## Author Contributions

SY and KN wrote the article and approved the submitted version.

## Conflict of Interest

The authors declare that the research was conducted in the absence of any commercial or financial relationships that could be construed as a potential conflict of interest.
